# Genome Sequences of Simian Hemorrhagic Fever Virus Variant NIH LVR42-0/M6941 Isolates (*Arteriviridae*: *Arterivirus*)

**DOI:** 10.1128/genomeA.00978-14

**Published:** 2014-10-09

**Authors:** Michael Lauck, Gustavo Palacios, Michael R. Wiley, Yànhuá Lǐ, Yīng Fāng, Matthew G. Lackemeyer, Yíngyún Caì, Adam L. Bailey, Elena Postnikova, Sheli R. Radoshitzky, Reed F. Johnson, Sergey V. Alkhovsky, Petr G. Deriabin, Thomas C. Friedrich, Tony L. Goldberg, Peter B. Jahrling, David H. O’Connor, Jens H. Kuhn

**Affiliations:** a University of Wisconsin-Madison, Madison, Wisconsin, USA; b United States Army Medical Research Institute of Infectious Diseases, Fort Detrick, Frederick, Maryland, USA; c Kansas State University, Manhattan, Kansas, USA; d Integrated Research Facility at Fort Detrick, National Institute of Allergy and Infectious Diseases, National Institutes of Health, Fort Detrick, Frederick, Maryland, USA; e Emerging Viral Pathogens Section, National Institute of Allergy and Infectious Diseases, National Institutes of Health, Fort Detrick, Frederick, Maryland, USA; f D. I. Ivanovsky Institute of Virology, Moscow, Russia

## Abstract

Simian hemorrhagic fever virus (SHFV) variant NIH LVR42-0/M6941 is the only remaining SHFV in culture, and only a single genome sequence record exists in GenBank/RefSeq. We compared the genomic sequence of NIH LVR42-0/M6941 acquired from the ATCC in 2011 to NIH LVR42-0/M6941 genomes sequenced directly from nonhuman primates experimentally infected in 1989.

## GENOME ANNOUNCEMENT

Simian hemorrhagic fever virus (SHFV), a member of the genus *Arterivirus* (*Nidovirales*: *Arteriviridae*), causes viral hemorrhagic fever (VHF) in captive Asian macaques (reviewed in reference 1). Only one variant, NIH LVR42-0/M6941, remains from previous outbreaks. This variant (ATCC VR-533) was derived in the 1960s from a moribund rhesus monkey (*Macaca mulatta*) inoculated with a whole blood suspension from a deceased SHFV-infected stump-tailed macaque (*Macaca arctoides*) (2). Results of several in-house unpublished experiments based on the only available genomic sequence of NIH LVR42-0/M6941 (GenBank accession numbers AF180391 and AF180391.1; RefSeq accession numbers NC_003092 and NC_003092.1) suggested that this sequence record might contain errors. Therefore, we acquired the NIH LVR42-0/M6941 isolate from the ATCC in 2011 and obtained the genomic sequence of the virus (KS_06_17_11) after passage on MARC-145 cells, a subclone of the MA-104 C-1 cell line (ATCC CRL-2378.1) (3). Another NIH LVR42-0/M6941-derived isolate (RJ_03_26_10) was maintained in MA-104 C-1 cell culture at the U.S. Army Research Institute of Infectious Diseases (USAMRIID) and the Integrated Research Facility at Fort Detrick (IRF-Frederick) for several years and used in a recently published nonhuman primate study (4).

Additionally, we generated six coding-complete SHFV genome sequences from twelve serum samples obtained from nonhuman primates experimentally infected with NIH LVR42-0/M6941 at USAMRIID in the late 1980s and early 1990s (1). These samples were obtained from two grivets (*Chlorocebus aethiops*; A095 and A230) and one rhesus monkey (I-618) on different days (d) postinoculation (A095_d5, A095-d7, A230_d5, A230_d7, I618_d3, and I-618_d5). Briefly, RNA was isolated with the omission of carrier RNA (Qiagen, Valencia, CA), and randomly primed double-stranded cDNA was synthesized (Life Technologies, Grand Island, NY), as previously described (5). Deep sequencing libraries were prepared using the Nextera XT kit (Illumina, San Diego, CA) and sequenced on the Illumina MiSeq. Using CLC Genomics Workbench 7.1 (CLC bio, Aarhus, DK), low quality (<q30) and short reads (<100 bp) were removed, and the remaining reads were mapped to NIH LVR42-0/M6941 (GenBank accession number AF180391; RefSeq accession number NC_003092).

We identified sequence discrepancies from comparison to sequences in the GenBank and RefSeq records. We detected the same 18 changes recently published and deposited (AF180391.2) by Vatter et al. (6). Furthermore, we also found three slight differences among the various isolates. At two sites, the RJ_03_26_10-infected cells and serum sequences differ from AF180391.2 (C → R and G → Q). Second, the ORF2a region of three genomes A095_d5, A095-d7, and A230_d7 are 11 codons shorter than that of AF180391.2. Finally, seven single nucleotide polymorphisms (SNPs) shared among genomic sequences from serum samples are absent in the KS_06_17_11 and RJ_03_26_10 samples. These slight differences may be the result of immune-mediated pressure *in vivo* during adaptation to a new host, since the natural host of the NIH LVR42-0/M6941 type strain remains unknown.

### Nucleotide sequence accession numbers.

The GenBank accession numbers of SHFV variant NIH LVR42-0/M6941 isolates KS_06_17_11, RJ_03_26_10, A095-d5, A095-d7, A230_d5, A230_d7, I618_d3, and I-618_d5 are KM373784, KM371111, KM371105, KM371106, KM371107, KM371108, KM371109, and KM371110, respectively.
